# Exploratory Machine Learning and Omics Integration in the Search for Biomarkers of Papillary Thyroid Cancer

**DOI:** 10.3390/biology15131004

**Published:** 2026-06-25

**Authors:** Pedro Henrique Godoy Sanches, Nicolly Clemente de Melo, Danilo Cardoso de Oliveira, Lucas Miguel de Carvalho

**Affiliations:** Health Sciences Postgraduate Program, São Francisco University, Bragança Paulista Campus, Av. São Francisco de Assis, 218, Bragança Paulista 12916-900, SP, Brazil

**Keywords:** papillary thyroid cancer, papillary thyroid carcinoma, bioinformatics, transcriptomics, metabolomics

## Abstract

Papillary thyroid carcinoma (PTC) is among the most common endocrine malignancies worldwide, and although generally associated with a favorable prognosis, a subset of patients develops aggressive disease with higher recurrence risk. This highlights the need for improved molecular characterization. Data integration approaches combined with computational methods offer new opportunities to refine diagnosis and uncover disease mechanisms. This study aims to integrate omics data and apply machine learning (ML) to identify clinically relevant biomarkers in papillary thyroid carcinoma. We selected 11 genes from the differentially expressed genes (DEGs)–LASSO intersection approach. Genes were validated using an independent external dataset (AUC = 91%, Sens. = 92%, Spec. = 97%, and Acc. = 95%). DEGs were integrated with metabolomics data from the literature, enabling the construction of a metabolite–gene interaction network, highlighting norepinephrine, arachidonic acid, and glutamic acid as representative metabolites, while the main genes were *SLC6A14*, *ADK*, *ATIC*, *NT5E*, and *AR*. We identified potential drug–gene interactions and performed survival analysis to assess the relevance of the possible biomarkers. This novel pipeline combining integration and machine learning provides new insights into thyroid cancer biology and identifies promising diagnostic markers, supporting advances in precision medicine.

## Simple Summary

Papillary thyroid carcinoma (PTC) is among the most common endocrine malignancies worldwide, and although generally associated with a favorable prognosis, a subset of patients develops aggressive disease with higher recurrence risk. This highlights the need for improved molecular characterization. This study aims to integrate multi-omics data and machine learning (ML) to identify clinically relevant biomarkers in papillary thyroid carcinoma. These models were validated using independent external datasets. Additionally, differentially expressed genes (DEGs) were integrated with metabolomics data from the literature, enabling the construction of a metabolite–gene interaction network. This novel pipeline combining data integration and machine learning provides new insights into thyroid cancer biology, supporting advances in precision medicine.

## 1. Introduction

Thyroid carcinoma (TC) ranks as the seventh most common malignancy worldwide, exhibiting a notable female predominance (3:1) and a higher incidence in developed countries [1]. Despite its high incidence, TC is generally associated with a favorable prognosis; however, a subset of patients presents with aggressive disease and increased risk of recurrence, highlighting the need for improved molecular characterization [2,3]. The histological landscape of TC is diverse: papillary thyroid carcinoma (PTC) accounts for approximately 80% of cases, followed by the follicular type (FTC) at 15%, while medullary (MTC) and anaplastic (ATC) carcinomas comprise 4% and 1% of occurrences, respectively [4].

Several risk factors have been proposed, including genetic, environmental, and hormonal influences, such as iodine intake, TSH level, autoimmune thyroid disease, gender, estrogen, obesity, lifestyle changes, and environmental pollutants, but up to now, only childhood exposure to ionizing radiation has been fully recognized as a risk factor [5,6]. Clinically, PTC is often characterized by slow tumor progression and nonspecific symptoms [3]. The main issue caused by PTC is excessive growth in size, compressing the surrounding regions, and causing mild symptoms. Thus, neck pain is the most reported symptom, and nerve compression can be an issue; respiratory difficulties are rarer [4].

Therapeutic strategies are currently guided by the American Thyroid Association’s (ATA) risk stratification, which classifies recurrence probability from low to high. Management typically involves surgical intervention via thyroidectomy, often complemented by adjuvant radioactive iodine therapy [7]. However, these approaches may lead to overtreatment in low-risk patients and remain insufficient for accurately predicting disease progression in more aggressive cases. In this context, recent advances in clinical management, including high-resolution imaging and molecular profiling, are reshaping the standard of care [8].

Omics sciences have emerged as a cornerstone of modern oncology, enabling the comprehensive characterization of molecular alterations at multiple biological levels [9], including transcriptomics [10], proteomics [11], and metabolomics [12]. These approaches have facilitated the identification of novel biomarkers and therapeutic targets across different cancer types [13]. In thyroid cancer, bioinformatics-driven studies have increasingly applied high-throughput data analysis to uncover gene signatures, regulatory networks, and diagnostic markers associated with PTC progression and prognosis [14,15,16]. Furthermore, the integration of omics layers with advanced computational approaches and artificial intelligence have shown great potential to improve tumor classification, risk prediction, and personalized treatment strategies [17].

Although several studies have explored transcriptomic alterations in PTC, most rely on single-layer analyses, which may overlook complex biological interactions underlying tumor development. Here, we advance beyond traditional approaches by integrating gene expression profiles with literature metabolomics data, enabling a systems-level understanding of the disease. In addition, the application of machine learning models allows for robust feature selection in high-dimensional datasets, reducing noise while preserving biologically relevant signals. The novelty of this strategy enhances biomarker discovery and improves model generalizability across independent cohorts, addressing a critical gap in translational oncology.

Additionally, as shown in a recent review [18], little has been done to provide clear biomarking features for PTC. While some decision tree models have been used to find patterns in lncRNAs and miRNAs, not much has been done to oversee the basic diagnostic criteria. In addition, no attempt has been made to integrate different omics layers. Our approach aims to identify clinically relevant biomarkers with medical value while also uncovering potential molecular mechanisms that could guide precision medicine strategies in papillary thyroid carcinoma.

## 2. Materials and Methods

We implemented an integrative multistep analytical framework combining transcriptomic profiling, machine learning, and clinical outcome assessment. Initially, we searched for repositories with publicly available experimental data on PTC for both metabolomics and transcriptomics. Transcriptomics studies were found in Gene Expression Omnibus (GEO) under the ID GSE29265 for the training set and GSE33630 for the validation set. The search for metabolomics data was conducted in Metabolomics Workbench [19] and Metabolights [20]. Unfortunately, there were no experimental data from metabolomics studies; thus, a manual curation was considered. To obtain publicly available data, a manual search in the PUBMED database was performed—the terms used were “Metabolomics” and “Papillary Thyroid Carcinoma”.

Firstly, the main inclusion criteria for a metabolite were the presence of an official name together with its fold-change. Secondly, the developed algorithm for transcriptomics data was applied. It consisted of: (i) data handling—log2 normalization, remove near zero variance; (ii) differential expression analysis—(using limma package) fit linear model, Bayes empirical moderation, Wilcoxon analysis (FDR < 0.05) and fold-change (|log2FC| > 0.58); (iii) Least Absolute Shrinkage and Selection Operator (*LASSO*) model—cross-validation (best λ), fit LASSO model, ROC curve to evaluate; and (iv) intersection—intersects limma’s DEGs with LASSO-selected features to find a final list of genes (*n* = 11).

**Figure 1:**
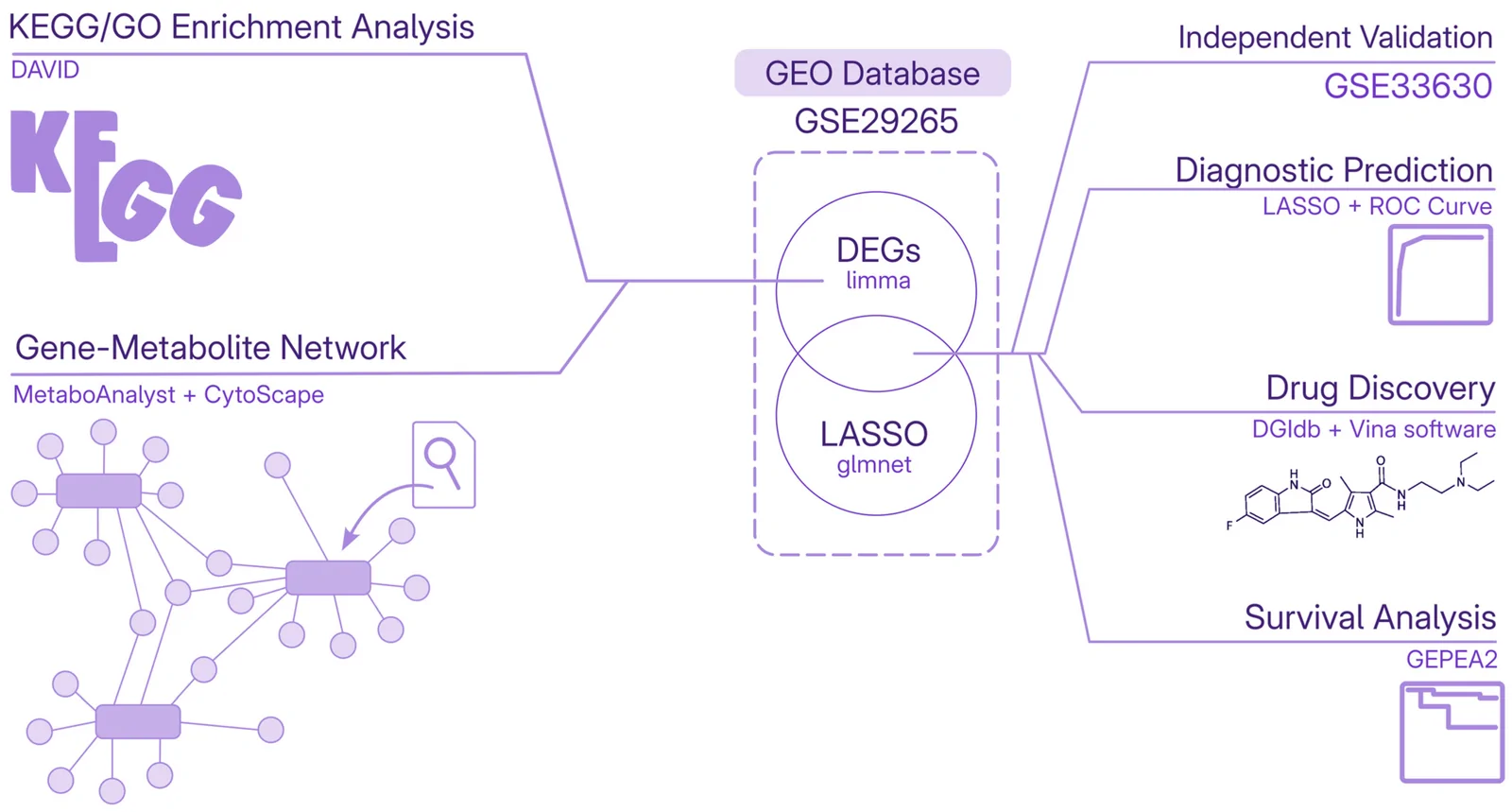
The whole pipeline developed to associate transcriptomics data with metabolomics data. Arrows indicate the direction of the analytical workflow, and boxes represent the main processing and analysis steps, including metabolite selection, gene identification, network construction, survival analysis, drug discovery, and enrichment analyses.

Finally, a network of interactions among genes and literature-gathered metabolites was built using MetaboAnalyst v6.0 [21] and exported to CytoScape v3.10.3 [22] where parameters of quality were calculated. Additionally, overall survival analysis and disease-free survival analysis were performed using the selected genes (*n* = 11). Afterward, in silico matching of the intersected genes with FDA-approved drugs was conducted with additional enrichment analysis. For both metabolomics and transcriptomics, enrichment analysis was also explored (Figure 1).

All analyses were conducted using fully reproducible results, which are provided in the Supplementary Material.

### 2.1. Datasets and Preprocessing

Gene expression datasets were obtained from the Gene Expression Omnibus (GEO) database. The dataset GSE29265 was used as the training cohort, comprising 20 papillary thyroid cancer (PTC) samples and 20 normal thyroid samples. The dataset GSE33630, containing 49 PTC and 45 normal samples, was used as an independent validation cohort. Expression data were assessed for scale, and log2 transformation was applied when gene expression value exceeded 50. Genes with near-zero variance (<1 × 10^−6^) in the training dataset were excluded. Principal component analysis (PCA) was performed to evaluate sample distribution and data quality.

### 2.2. Differential Expression Analysis

Differential expression analysis between PTC and normal samples in the training dataset (GSE29265) was performed using the limma package (v3.66.0) [23]. A linear model was fitted to the data, followed by empirical Bayes moderation. Genes were considered differentially expressed based on the following criteria: adjusted *p*-value (false discovery rate, FDR) < 0.05 and absolute log2 fold change (|log2FC|) > 0.58.

### 2.3. LASSO Feature Selection

To identify predictive features, a LASSO logistic regression model was implemented using the glmnet package (v5.0) [24]. To reduce dimensionality and computational burden, the top 5000 most variable genes were selected. A binomial model was applied, and 5-fold cross-validation was used to determine the optimal regularization parameter (λ). Genes with non-zero coefficients in the final LASSO model were selected as relevant features.

### 2.4. Intersection Strategy and External Validation

To increase robustness, an intersection strategy was applied by overlapping the list of differentially expressed genes (DEGs) with the genes selected from LASSO. The resulting intersecting genes were considered candidate biomarkers. These genes were used to build a predictive model, which was evaluated in the independent validation dataset (GSE33630). Only genes present in both datasets were retained.

Prediction probabilities were calculated using the LASSO-derived coefficients. Model performance was assessed by a receiver operating characteristic (ROC) curve analysis, and the area under the curve (AUC), sensitivity, specificity, and accuracy were calculated. The optimal classification threshold was determined based on ROC curve coordinates. Additionally, statistical differences in gene expression between PTC and normal samples in the validation dataset were assessed using the Wilcoxon rank-sum test (*p*-value < 0.05).

### 2.5. Survival and Progression-Free Interval Analysis

Overall survival and progression-free interval analysis were performed using the GEPIA3 platform [25], based on The Cancer Genome Atlas (TCGA) thyroid carcinoma dataset. Genes identified as statistically significant were evaluated for their association with patient outcomes.

Overall survival and disease-free survival analyses were conducted by stratifying patients into high- and low-expression groups according to gene expression levels. Kaplan–Meier survival curves were generated, and statistical significance was assessed using the *p*-value (*p* < 0.05).

### 2.6. Drug–Gene Interaction Analysis

In order to generate hypotheses and find possible links between genes and drugs, we here use the DGIdb [26] as a publicly available drug–gene interaction platform, where the 11 genes found in the Venn diagram were imported into the platform. We only selected drugs that were approved by the FDA.

### 2.7. Functional Enrichment Analysis

To investigate the biological functions and pathways associated with the initial 1428 differentially expressed genes (DEGs), Kyoto Encyclopedia of Genes and Genomes (KEGG) pathway and biological process in Gene Ontology (GO) enrichment analyses were performed using the Database for Annotation, Visualization, and Integrated Discovery (DAVID) platform [27], considering *p*-adj < 0.05 as a significance cutoff.

### 2.8. Metabolites Associated with PTC

To investigate metabolite associations with PTC, datasets from published metabolomics studies focusing on papillary thyroid cancer were collected from the PUBMED database using the terms “Papillary Thyroid Carcinoma” AND “Metabolomics”. At the time of the systematic search, 58 articles were found and filtered by title (*n* = 33), considering the correct study design (in this case, PTC vs. nontumoral). Additionally, filtering by the abstract and text (*n* = 28) was performed, and finally, by data availability (*n* = 7).

Metabolomics is a younger field compared to transcriptomics; thus, it is expected from the articles not to have much publicly available data. In this case, a manual curation was necessary, searching for the presence of a link, corresponding author email, public repository upload, or other ways of data availability. In the case of manual collection of data, the presence of a reported *p*-value and fold-change were mandatory (*n* = 3).

### 2.9. Integration Strategy

An integrative approach was applied to combine transcriptomic and metabolomic data [28]. Differentially expressed genes identified in the training dataset were integrated with PTC-associated metabolites. Gene–metabolite interaction networks were constructed using the MetaboAnalyst platform [21].

Network topology analysis was performed to identify key nodes using CytoHubba in Cytoscape software [22], and the top three genes and top three metabolites with the highest degree of centrality were selected as hub components. This integrative strategy aimed to uncover potential molecular mechanisms linking gene expression and metabolic alterations in papillary thyroid cancer.

### 2.10. Molecular Docking

The molecular docking approach was applied to analyze the receptor–ligand relationship. The genes used for docking were selected from the integration between DEGs and LASSO. Given the challenges and costs of antibody production, only small molecules were selected to evaluate drug repurposing in this study, due to their potential to complement and synergize with therapeutics compared with antibody-based immunotherapy [29].

The Protein Data Bank (PDB) [30] was used to curate experimentally resolved 3D protein structures based on the presence of small-molecule ligand information. Homo sapiens were the source organism, refinement resolution between 2 and 3 Å, and 100% amino acid sequence identity. The amino acid sequence was retrieved from KEGG by searching *Homo sapiens* species in the “Gene” section. Protein and ligand preparation followed these steps: AutoDock Vina v1.2.x [31] was used to perform molecular docking, and the AutoDockTools (ADT) v1.5.7 [32] graphical interface to prepare the protein at a pH of 7.4 with Kollman charges. In order to prepare the ligands, we combined RDKit [33] and OpenBabel [34] to convert molecules to “pdbqt” files and correct hydrogen states.

In cases where the gene did not have an inhibitor in the PDB file, a blind docking approach was performed. The predicted structure was retrieved from UniProt [35] by AlphaFold [36]. The KVFinder-web server [37] was used to predict cavities and potential active site regions. Discovery Studio (DS) v24.1.0.23298 [38] was utilized to identify the coordinates of active sites of the proteins in docking and blind docking.

The generated files were visualized using UCSF ChimeraX v1.8 [39], and amino acid interactions were analyzed in DS. Uniprot [40] was used to confirm amino acid positions in the predicted binding site using gene symbols for searching. The results with the best binding energy rankings were qualified, with more negative values indicating higher affinity between the ligand and the protein, representing a more favorable and stable binding to the target.

## 3. Results

### 3.1. Combined Differential Expression and Feature Selection Pipeline

**Figure 2:**
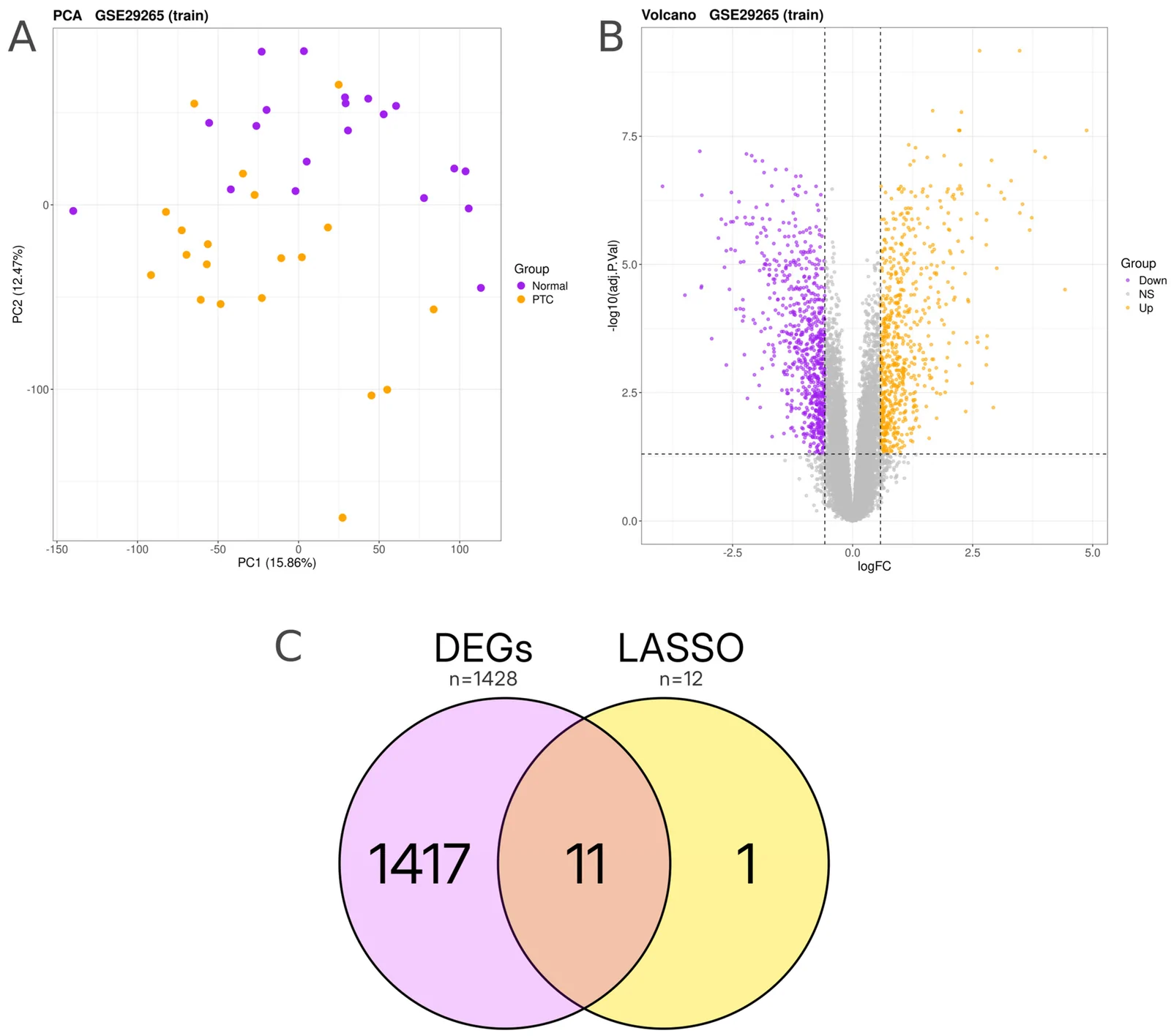
In (**A**), principal component analysis displays nontumoral tissue in purple, while papillary thyroid carcinoma is in orange. Likewise, (**B**) shows a volcano plot, where the purple dots represent the downregulated variables while orange the upregulated ones; the nonsignificant (NS) features are displayed in gray; dashed line represent the significance limits. The Venn diagram in (**C**) shows the left circle, the differentially expressed genes (DEGs), intersecting with the LASSO model (Least Absolute Shrinkage and Selection Operator).

After preprocessing of the GSE29265 dataset, a total of 23,520 genes were retained for downstream analysis following probe collapsing and variance filtering. Principal component analysis (PCA) revealed a clear separation between PTC and normal samples, indicating strong transcriptomic differences between the groups (Figure 2A). Differential expression analysis identified 1428 differentially expressed genes (DEGs) (adjusted *p*-value < 0.05 and |log2FC| > 0.58), of which 679 were upregulated and 749 were downregulated in PTC samples (Figure 2B and Supplementary Table S1).

The LASSO model, applied to the training dataset, selected 12 genes with non-zero coefficients under the optimal λ = 0.098 determined by cross-validation (Supplementary Table S2). These genes represent features with the highest predictive power for distinguishing PTC from normal samples.

The intersection between DEGs and LASSO-selected genes resulted in 11 genes (Figure 2C) which were considered robust candidate biomarkers, namely *DPP4*, *GLT1D1*, *TENM1*, *TIMP4*, *PDZK1IP1*, *ABHD17C*, *IGSF10*, *RHOU*, *PDGFC*, *ABCA6*, and *RPS16P5*.

### 3.2. Validation of the Selected Biomarkers

The predictive performance of the 11 intersecting genes was evaluated in the independent GSE33630 dataset. The model achieved an area under the curve (AUC) of 91%, indicating strong discrimination between PTC and normal samples. Using the optimal threshold derived from ROC analysis, the model achieved an area under the curve (AUC) of 91%, sensitivity of 92%, specificity of 97%, and overall accuracy of 95%.

**Figure 3:**
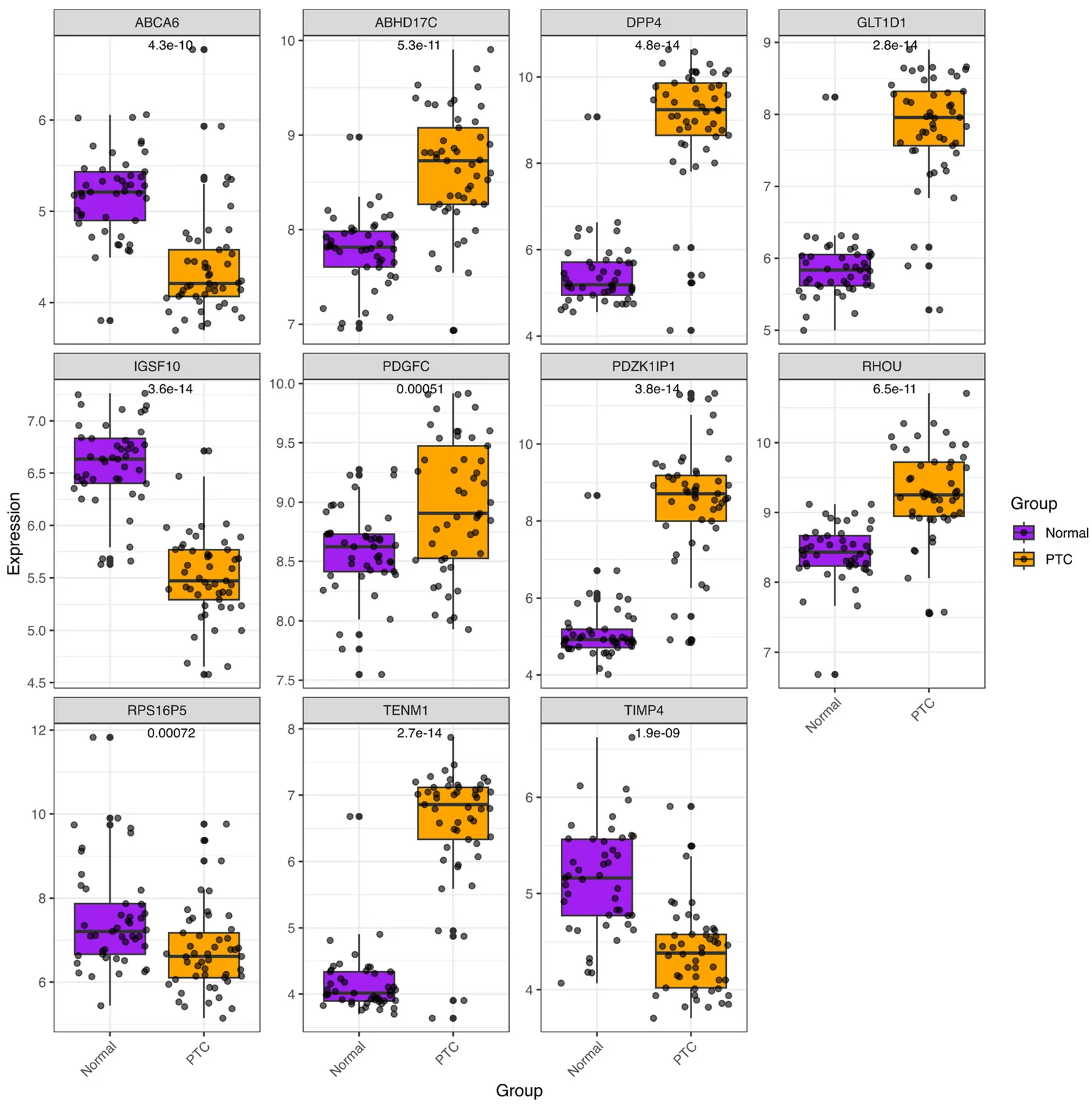
All the boxplots for the selected genes. Wilcoxon’s adjusted *p*-value is shown above the boxes. The *y*-axis expresses the log2 (Expression), while the *x*-axis displays the compared groups. Purple boxes show the nontumoral (“normal”) tissues and the orange ones the papillary thyroid carcinoma. At the top of the plot’s panel is displayed the Wilcoxon’s significance value corrected by the false discovery rate (*ABCA6* (*p* = 4.3 × 10^−10^), *ABHD17C* (*p* = 5.3 × 10^−11^), *DPP4* (*p* = 4.8 × 10^−14^), *GLT1D1* (*p* = 2.8 × 10^−14^), *IGSF10* (*p* = 3.6 × 10^−14^), *PDGFC* (*p* = 5.1 × 10^−4^), *PDZ1IP1* (*p* = 3.8 × 10^−14^), *RHOU* (*p* = 6.5 × 10^−11^), *RPS16P5* (*p* = 7.2 × 10^−4^), *TENM1* (*p* = 2.7 × 10^−14^), and *TIMP4* (*p* = 1.9 × 10^−9^)).

Statistical analysis demonstrated that the selected genes maintained consistent expression patterns in the validation dataset, with 11 out of the 11 genes meeting the Wilcoxon significance threshold (*p* < 0.05) and showing significant differences between PTC and normal samples (Figure 3). These findings support the reproducibility and biological relevance of the identified biomarkers.

There were four downregulated genes (*ABCA6*, *IGSF10*, *RPS16P5*, and *TIMP4*) and seven upregulated genes (*ABHD17C*, *DPP4*, *GLT1D1*, *PDGFC*, *PDZK1IP1*, *RHOU*, and *TENM1*). There was no strong evidence in the literature on the function of *GLT1D1*, *IGSF10*, and *RPS16P5*. However, *DPP4*, *TENM1*, *TIMP4*, *PDZK1IP1*, *RHOU*, *PDGFC*, and *ABCA6* return some relationships with PTC, organized individually in Supplementary Table S3. The individual statistical and prediction values are displayed in Table 1.

### 3.3. Overall Survival and Progression-Free Interval Analyses

**Figure 4:**
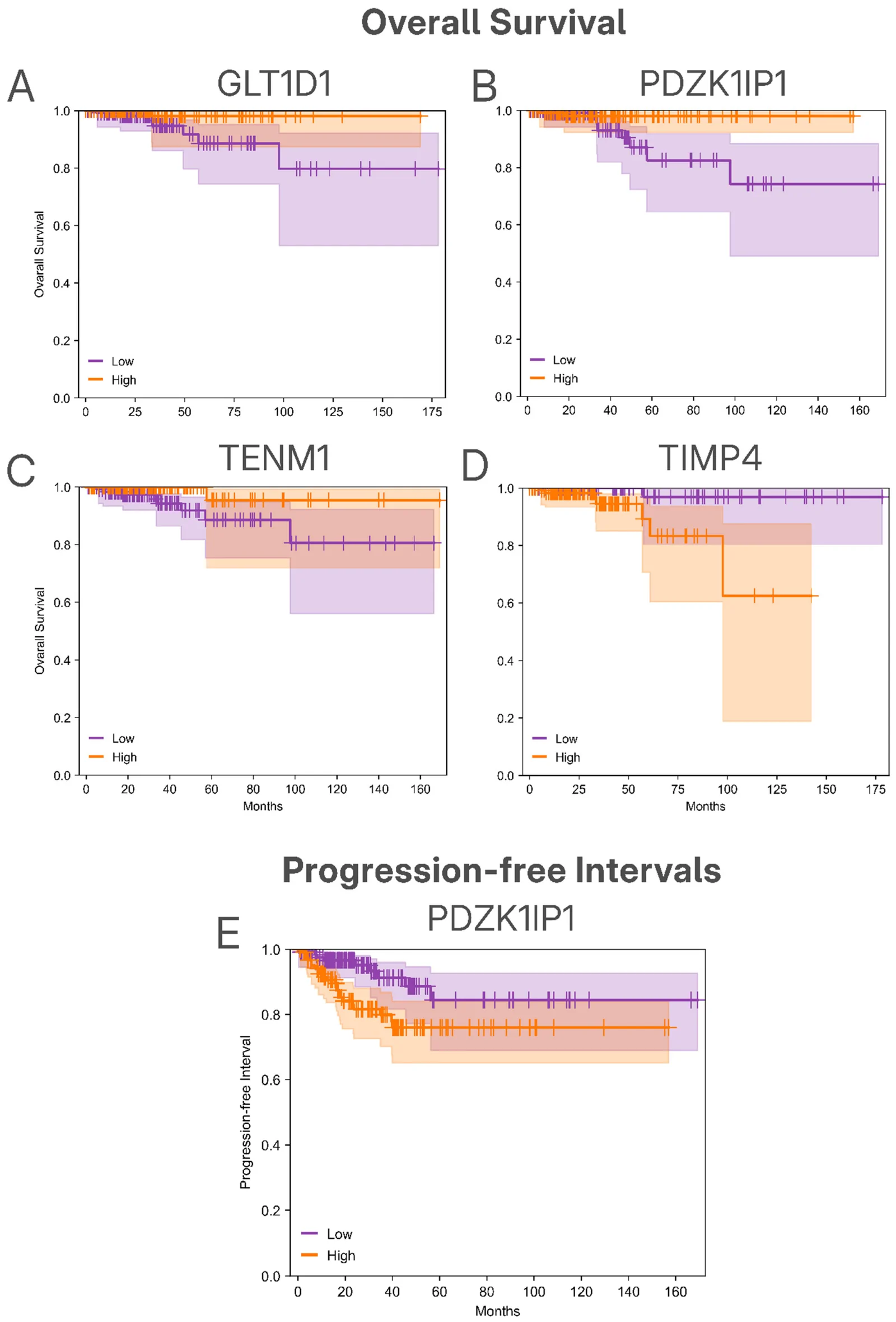
Overall survival curves in (**A**) are displayed for the *GLT1D1* gene (*p* = 0.0494), in (**B**), the *PDZK1IP1* gene (*p* = 0.0398), in (**C**), the *TENM1* gene (*p* = 0.036), and (**D**), the *TIMP4* gene (*p* = 0.00507). The progression-free intervals in (**E**) show the gene *PDZK1IP1* with *p* = 0.0145. The orange lines represent the high group and the purple one the low group. Confidence intervals are shown around the line as a painted box.

Overall survival analysis using TCGA data revealed that four of the selected genes were significantly associated with patient outcomes (*GLTD1*, *PDZK1IP1*, *TENM1*, and *TIMP4*). Kaplan–Meier curves demonstrated that altered expression levels of these genes were linked to differences in both overall survival (OS) (Figure 4A–D; Supplementary Table S4) and progression-free intervals (Figure 4E; Supplementary Table S5). Specifically, patients stratified into high- and low-expression groups showed significantly distinct survival probabilities (*p* < 0.05), indicating that these genes may have prognostic value in PTC.

### 3.4. Enrichment Analysis of DEGs

Functional enrichment analysis of DEGs revealed significant involvement of these genes in PTC (Supplementary Table S6). Several pathways were returned, including cytoskeleton in muscle cells (FDR = 0.0000617), cell adhesion molecules (FDR = 0.00525), cytokine–cytokine receptor interaction (FDR = 0.00382), but also transcriptional misregulation in cancer (FDR = 0.00292), the p53 signaling pathway (FDR = 0.00292), and extracellular matrix–receptor interaction (FDR = 0.00292). Table 2 compiles the GO biological process enrichment analysis.

### 3.5. Molecular Docking of the Drug Interactions

The gene–drug interaction analysis performed using DGIdb identified two genes with FDA-approved associated drugs: *PDGFC* and *DPP4* (Supplementary Table S7). In total, 22 drugs were retrieved for these genes. However, when restricting the analysis to compounds classified as antineoplastic agents, only a limited subset of interactions remained. After these selections, we performed molecular docking to confirm this promising interaction only for small molecules (see Section 2.10).

For PDGFC, a single approved antineoplastic agent was identified, Sunitinib, with an interaction score of 1.0876. Cavities were predicted using KVFinder that revealed two pockets named KAK and KAD due to its homodimeric structure. After that, Discovery Studio was used to determine the docking-grid coordinates (KAD: X = −8.597717, Y = 16.791843, Z = 3.858157; KAK: X = 3.858157, Y = −12.895208, Z = −14.675940).

**Figure 5:**
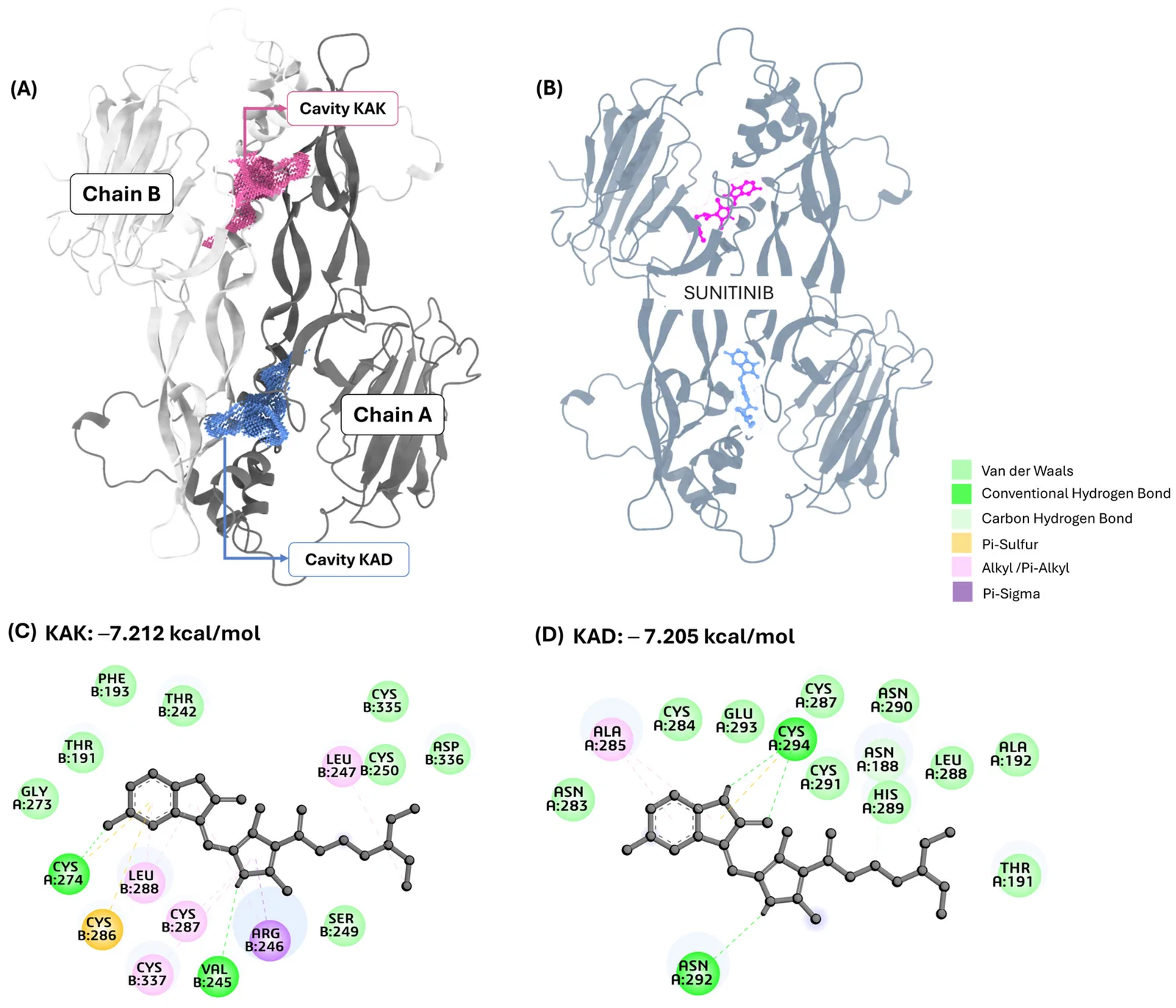
Blind docking of Sunitinib with PDFCG after cavity prediction. (**A**) 3D mapping of KAK (pink) and KAD (blue) cavities predicted by KVFinder-web in PDFCG predicted by AlphaFold constructed in two chains. (**B**) Predicted binding poses of Sunitinib in both protein pockets. (**C**) 2D interaction map for the KAK site (−7.212 kcal/mol); (**D**) 2D interaction map for the KAD site (−7.205 kcal/mol). Each interaction type color is represented in the figure.

Blind docking analysis revealed that Sunitinib binds favorably to both predicted cavities (KAK and KAD) of PDGFC, with similar binding affinities (−7.212 and −7.205 kcal/mol, respectively), indicating strong affinity across the identified binding sites (Figure 5).

For *DPP4*, several approved antineoplastic agents were identified. These include Nintedanib esylate (interaction score: 0.0409), Masitinib (0.0602), Quizartinib (0.0539), and Olaratumab (0.5118). Among these, Olaratumab showed the highest interaction score for *DPP4* but was not selected for docking tests, as it is an antibody, and thus was not included in the analysis.

**Figure 6:**
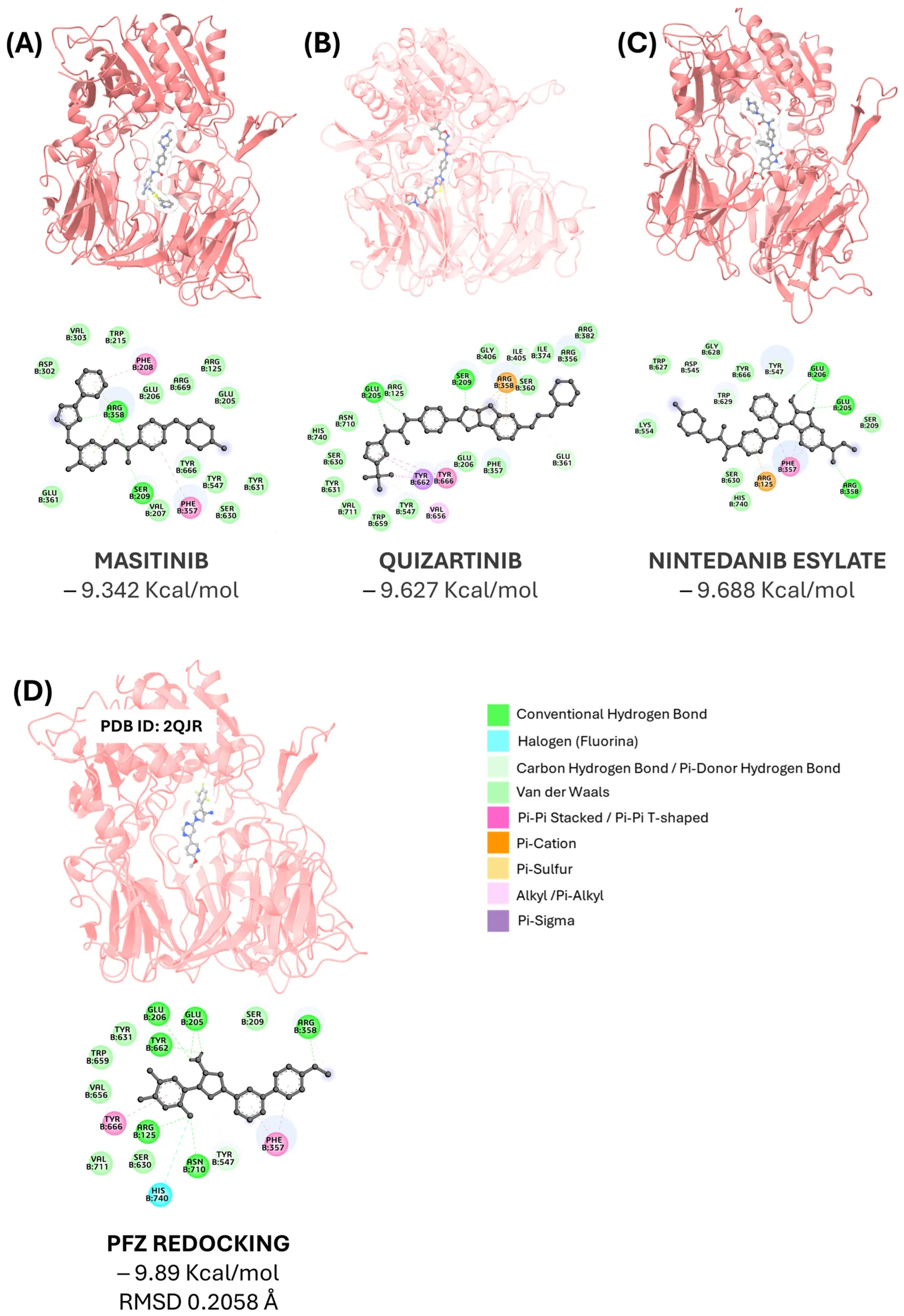
Molecular docking results between selected drugs and the DPP4 protein. (**A**) Docking result of Masitinib with a binding energy of −9.342 kcal/mol; the 3D structure is positioned within the active site, featuring a prevalence of Van der Waals and conventional hydrogen bond interactions. (**B**) Quizartinib presented a binding energy of −9.627 kcal/mol; transparency was applied to demonstrate a minor deviation of the molecule from the active site, with interactions primarily consisting of Van der Waals and conventional hydrogen bonds. (**C**) Docking result for Nintedanib esylate with −9.688 kcal/mol of energy; the molecule is in the active site, showing a predominance of Van der Waals and conventional hydrogen bond types. (**D**) Redocking results for (3R,4S)-1-[6-(6-methoxypyridin-3-yl)pyrimidin-4-yl]-4-(2,4,5-trifluorophenyl)pyrrolidin-3-amine (PZF) showed a binding affinity of −9.89 kcal/mol; interactions included Van der Waals, conventional hydrogen bonds, and exclusive halogen bond types. Each interaction type color is represented in the figure.

The binding site was set at X = −1.775483, Y = −5.364172, Z = 72.346828 after identifying the native ligand coordinates in the PDB file. Molecular docking analysis demonstrated that Masitinib, Quizartinib, and Nintedanib esylate exhibit strong binding affinities to DPP4 (−9.342, −9.627, and −9.688 kcal/mol, respectively), comparable to the reference ligand PZF (−9.89 kcal/mol), with interactions predominantly mediated by Van der Waals forces and conventional hydrogen bonds within the active site. The active site of DPP4 can be defined by key amino acid residues at positions 630, 670, and 740, of which at least one is also involved in the interactions observed for the analyzed candidates (Figure 6).

### 3.6. Data Integration and Network Analysis of PTC

A total of 58 metabolomics studies focusing on PTC were curated, from which 101 metabolites reported as significantly altered were compiled (Supplementary Table S8). These metabolites were integrated with the DEGs identified in the transcriptomic analysis.

A gene–metabolite interaction network was constructed, revealing complex relationships between deregulated genes and altered metabolites (Supplementary Figure S1). Network topology analysis identified key hub components, with the top three genes (*SLC6A14*, 8; *ADK*, 8; *ATIC*, 5) and top three metabolites (norepinephrine, 24; arachidonic acid, 23; glutamic acid, 21) showing the highest degree of centrality.

For a more profound description of the interactions, Table 3 summarizes the connections made from the genes to the metabolites.

**Figure 7:**
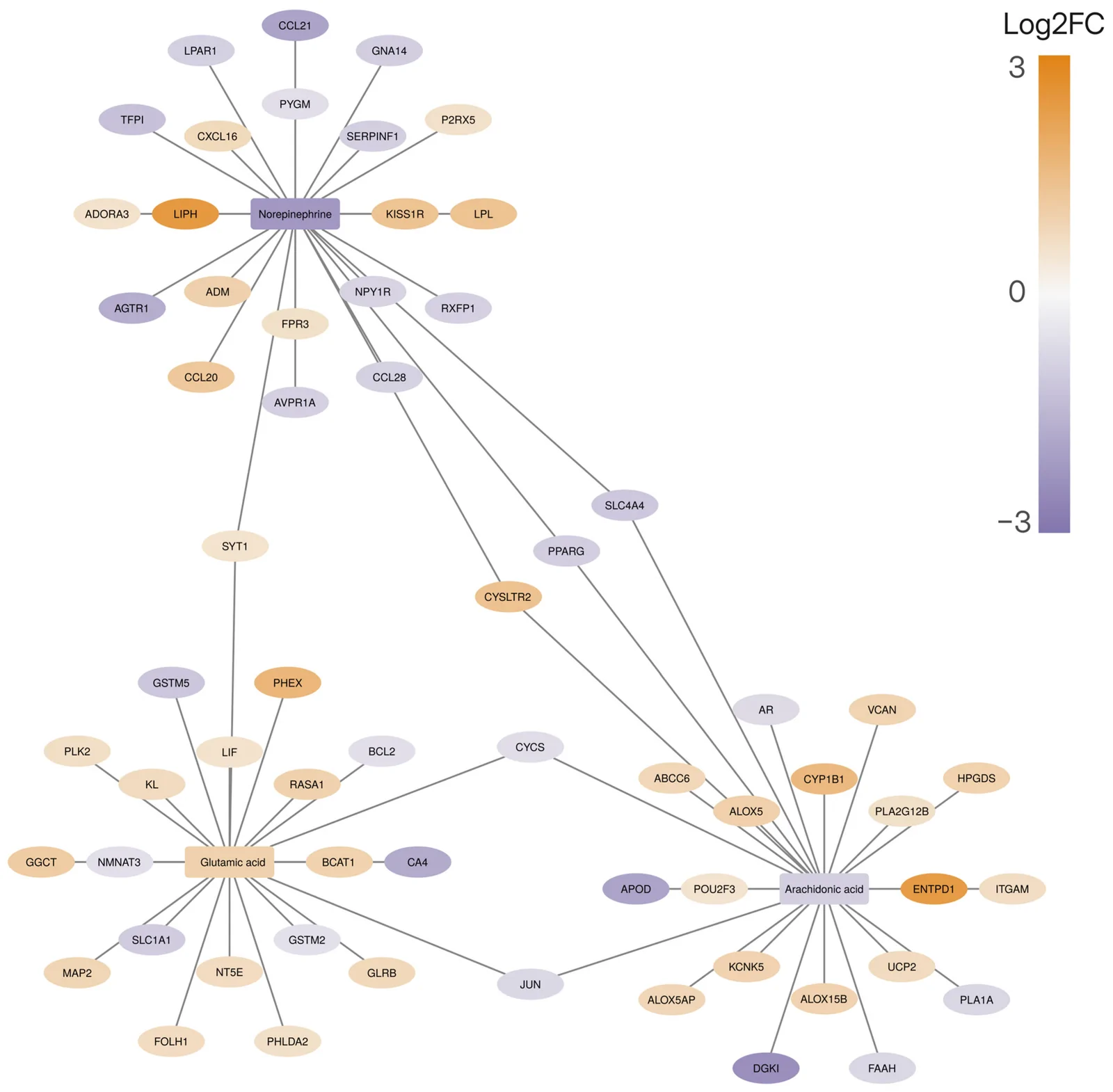
Gene–metabolite network analysis, showing the top three metabolites. The squared shapes represent metabolites, while the circles represent genes. The scale at the right shows the log2FC scale, where each component is then colorized accordingly.

Additionally, the top three metabolites’ networks, shown in Figure 7, display several DEGs connected to the main metabolites, but some highlight the interaction of the nodes: *SYT1* (FDR = 0.0046) connects norepinephrine to glutamic acid, while *CYSLTR2* (FDR = 3.9195 × 10^−6^), *PPARG* (FDR = 0.0007), and *SLC4A4* (FDR = 3.0020 × 10^−5^) link norepinephrine to the arachidonic acid. Finally, *CYCS* (FDR = 0.0031) and *JUN* (FDR = 0.0104) connect glutamic acid to arachidonic acid, rendering those genes as potential regulators of the interaction among these metabolites.

## 4. Discussion

### 4.1. Biological Significance of the Selected Biomarkers

It was possible to select variables beyond DEGs, intersecting them with advanced machine learning models (namely, LASSO). Some genes (*DPP4*, *GLT1D1*, *PDZK1IP1*, and *TENM1*) displayed a distinct separate boxplot (Figure 3), indicating more considerable differences between PTC and normal tissue, thus indicating that a hyperexpression status was active and could contribute to the disease’s carcinogenesis. Particularly, *PDZK1IP1* showed a considerable separation in the boxplot and was shown as significant in the survival analysis, indicating that it could represent a prognostic feature, discussed further in more detail.

Other work [41] has found ABCA6 as a hub gene in their analysis, reaching AUC = 89.5%. They used several datasets, including the one used here as an independent data validation (*GSE33630*), thus explaining why it appeared. Here, we selected *ABCA6* by the intersection approach; nonetheless, the gene did not show up as a hub gene. Their technique was network-based, and as both works used different approaches, the *ABCA6* gene could actually have an important role in PTC by being selected.

*DPP4* was identified among the genes selected by the LASSO model and showed increased expression in PTC compared with normal thyroid tissues. The marked differential expression observed in our dataset supports its potential relevance in PTC biology. It functions as a peptidase involved in chemokine processing, signal transduction, and glucose metabolism [42]. Previous studies have reported that elevated *DPP4* expression is associated with poor prognosis in thyroid cancer and that its inhibition may affect pathways related to tumor progression and resistance to programmed cell death [43].

*PDGFC* was identified among the selected biomarkers and showed increased expression in PTC samples. Although it was not associated with survival in our cohort, previous studies have reported an inverse correlation between *PDGFC* expression and lymphocyte infiltration in PTC tissues [44]. This observation may suggest a role in influencing the tumor microenvironment and could help explain its selection among the most informative genes. Additionally, in this work, we found the gene to be associated with an antineoplastic agent, namely, Sunitinib.

*TENM1* was upregulated in PTC samples compared with normal thyroid tissues in our analysis. Furthermore, *TENM1* expression was significantly associated with overall survival, supporting its potential prognostic relevance. This expression pattern is consistent with previous studies reporting increased *TENM1* expression in thyroid cancer. Prior evidence has suggested a possible involvement of *TENM1* in angiogenesis and tumor progression, which may provide a biological explanation for the dysregulation observed in our dataset [45].

*PDZK1IP1* exhibited expression differences between PTC and normal tissues and was significantly associated with both overall survival and progression-free intervals in our cohort, highlighting its potential prognostic utility. Previous enrichment analyses have linked *PDZK1IP1* to neutrophil activation and other immune-related pathways in PTC [46].

*GLT1D1* was identified among the biomarkers selected by the integrative DEG–LASSO approach and was significantly associated with overall survival in our cohort, suggesting potential prognostic relevance in PTC. Although the specific role of *GLT1D1* in thyroid cancer remains poorly characterized, previous studies have linked this gene to tumor immune evasion mechanisms. In lymphoma models, *GLT1D1* overexpression promoted tumor growth by increasing *PD-L1* glycosylation, thereby facilitating immune escape, whereas *GLT1D1* downregulation reduced glycosylated *PD-L1* levels and enhanced cytotoxic T-cell activity [47].

Additionally, post-translational modifications have been implicated in *PD-L1* regulation in other malignancies, with succinylation being associated with increased *PD-L1* expression in prostate cancer [48]. Together, these findings suggest that *GLT1D1* may contribute to pathways involved in *PD-L1* regulation and tumor–immune interactions.

*IGSF10* was identified among the candidate biomarkers selected, and its expression is reduced in PTC samples. Although the biological role of *IGSF10* in thyroid cancer remains poorly understood, accumulating evidence suggests that it may be involved in tumor progression and immune-related processes. A pan-cancer analysis demonstrated that *IGSF10* expression was associated with patient prognosis and showed strong correlations with immune cell infiltration, immune checkpoints, and several immunomodulatory factors [49]. In addition, as we observed in PTC samples, reduced *IGSF10* expression has been reported in breast cancer, where lower expression levels were associated with shorter overall and relapse-free survival [50].

*RPS16P5* had a decreased expression in thyroid tissues in our validation analysis. It is a retrocopy (processed pseudogene) belonging to the ribosomal protein S16 gene family, whose biological function remains largely uncharacterized. Previous transcriptomic analyses have reported subtype-specific expression patterns of retrogenes across different cancers.

In breast cancer, *RPS16P5* was found to be differentially expressed in the triple-negative subtype among a set of retrogenes exhibiting distinct regulation patterns associated with tumor subtype specificity [51]. Although functional evidence for *RPS16P5* is still lacking, retrogenes have been increasingly recognized as potential regulators of gene expression and tumor biology, and their differential expression has been associated with malignant phenotypes in several cancer types [51]. However, the specific role of RPS16P5 in thyroid cancer remains unknown and requires further investigation.

### 4.2. Survival Analyses of the Selected Genes

The most noticeable difference (*p* = 0.00507) lies in *TIMP4* (Figure 4D), which, according to Kaplan–Meier survival curves, the higher the expression, the worse the outcome. It regulates carcinogenesis through enriching the stem cell population in cervical cancer cells, such as the thyroid itself, giving it volume [52]. Additionally, *TENM1* (*p* = 0.036) (Figure 4C) was already reported in PTC associated with the MAPK signaling pathway [45], reported to be responsible for cell proliferation, differentiation, migration, senescence, and apoptosis [53].

Changes in the RAS-MAPK pathway have been reported in human cancer, including PTC, with abnormal behavior mainly in the RAS or RAF proteins (associated with the development of thyroid carcinomas) [54]. Additionally, the *BRAF* gene is also a component of the MAPK cascade of reactions and has also been reported in PTC. Strategies focusing on using the RAS-MAPK pathway as a therapeutic target have already been discussed in the literature [55].

The only progression-free interval curve that returned significant value was the *PDZK1IP1* curve, with *p* = 0.0398. It acts as an oncogene and modulates the tumor immune microenvironment on PTC [46], but also can be related to the proliferation, migration, invasion, apoptosis, and cell cycle arrest of PTC cells [56].

Additionally, the gene was overexpressed in the tumoral group (Figure 3), indicating its potential as a biomarker. However, this observation should be taken with a grain of salt due to a conflicting result: the overall survival curve indicates that the lower the expression, the worse the outcome, whereas in the progression-free interval curve, the higher the expression, the worse the outcome.

### 4.3. Biological Interpretation of Enriched Pathways

The pathway returned spans basic cellular function and organization to regulatory functions. The cytoskeleton in muscle cells and cell adhesion molecules provide support to cancer to thrive, favoring cell–cell adhesion, which can considerably increase its size, leading to symptoms like local compression.

On the other hand, it can modulate inflammatory response, such as through cytokine–cytokine receptor interaction, as well as through tumor-related pathways (e.g., transcriptional misregulation in cancer and the p53 signaling pathway) and signaling mechanisms (extracellular matrix–receptor interaction), as compiled in Supplementary Table S6.

The enrichment analysis reveals a profound reorganization of cellular architecture and its interface with the tumor microenvironment. The enrichment of pathways related to ECM–receptor interaction, cell adhesion, and extracellular matrix organization indicates that the DEGs are strongly associated with the Epithelial–Mesenchymal Transition (EMT). It is a process where epithelial cells lose their tight cell–cell contacts and polarity and acquire mesenchymal traits that promote movement and invasion [57]. EMT contributes to cancer dissemination by downregulating epithelial markers and upregulating mesenchymal/ECM-remodeling genes, enabling invasion and survival in the microenvironment [58].

This process is fundamental to thyroid cancer progression, as it enables follicular cells to lose their polarity and cohesion, which is partly mediated by changes in homophilic cell–cell adhesion, and acquire a migratory and invasive phenotype [59,60]. Furthermore, the enrichment of angiogenesis and ephrin receptor signaling corroborates the tumor’s capacity to remodel the local vasculature, facilitating metastatic dissemination to cervical lymph nodes—a common mark of aggressive PTC [61,62,63].

Simultaneously, the prominence of the inflammatory response reflects the intimate relationship between the immune microenvironment and thyroid oncogenesis [64]. The tumor appropriates these mechanisms to recruit immune cells that, rather than providing surveillance, secrete cytokines that promote proliferation and further tissue remodeling [64,65,66].

This chronic inflammatory state is often linked to the hydrogen peroxide catabolic process. While normal thyroid cells produce H_2_O_2_ for hormone synthesis, the dysregulation of this system in cancer leads to oxidative stress that activates survival cascades like PI3K/Akt signaling [67,68].

This is coupled with a marked loss of follicular identity, where thyroid hormone generation is downregulated as the cell redirects its energy toward cell population proliferation and fatty acid transport [69]. The metabolic shift promotes rapid growth and marks the transition toward radioiodine-refractory tumors, representing a significant challenge in clinical management.

### 4.4. Drug Interaction with Selected Biomarkers

Several proteins have already been described as therapeutic molecular targets for thyroid cancer, with response rates between 6% and 89%. Among these targets are *PDGFR*, *RET*, *KIT*, and others [70]. In this context, many studies have used molecular docking to identify new compounds targeting these receptors, as well as to evaluate potential ligands for novel proposed therapeutic targets. Studies that evaluated the affinity of new compounds or validated docking protocols through redocking of the RET protein, for example, reported binding energies around −8 kcal/mol, guiding the authors to conclude that the investigated compounds, potential anticancer agents, may inhibit pRET tyrosine kinase activity, although further in vitro and in vivo validation is needed [71].

Another study assessing the interaction of compounds with the DPP4 protein reported docking scores between −7 and −8 kcal/mol; the authors concluded that molecular docking analysis demonstrated promising binding affinities of sitagliptin toward the evaluated oncogenes, exceeding those observed for the reference inhibitors, making it a potential therapeutic option [72].

Overall, these reports support the results obtained in our study in an exploratory and hypothesis-generating context. Binding energies ranging from −7 to −9 kcal/mol, combined with favorable interactions between the ligand and key amino acid residues of the target protein, suggest that the investigated compounds in our research are promising candidates for further research and development as potential therapies for thyroid cancer.

Sunitinib is approved for the treatment of imatinib-resistant gastrointestinal stromal tumors (GISTs), renal carcinoma, and pancreatic neuroendocrine tumors, and as an oral tyrosine kinase inhibitor (TKI), it inhibits several receptors in the body, including important receptors for PTC, such as PDGF-R, VEGFR, FLT3, and RET, reducing tumor vascularization, which can reduce tumor size [73].

Nintedanib esylate is a triple angiokinase inhibitor that simultaneously blocks VEGFR 1–3, FGFR 1–3, and PDGFRα/β receptors, conferring particular biological relevance in the context of PTC. In PTC, these three receptors are frequently overexpressed, contributing to both tumor angiogenesis and the maintenance of the neoplastic microenvironment, including pericytes and smooth muscle cells involved in tumor vascularization [74].

Based on this molecular rationale, Nintedanib was evaluated in the randomized, double-blind, placebo-controlled phase II study EORTC-1209 (NCT01788982) [74,75]. Although the study did not demonstrate a clinically significant improvement in progression-free survival over the placebo, the triple inhibition profile of Nintedanib distinguishes it from the more selective approved inhibitors for this indication, such as Lenvatinib and Sorafenib, and may be of interest in specific molecular subgroups harboring *FGFR* or *PDGFR* amplification, targets that remain largely unexplored in standard therapies for advanced PTC. Importantly, Nintedanib esylate has been evaluated across multiple tumor types, where it has demonstrated favorable clinical outcomes [76,77].

Masitinib is an orally administered tyrosine kinase inhibitor primarily targeting the c-Kit receptor, while also inhibiting *PDGFRα/β*, *FAK*, *Lck*, and, to a lesser extent, *FGFR3* and *CSF1R* [78]. In the context of PTC, two of these targets have well-established and directly relevant molecular justifications. *PDGFRα*, in particular, plays a documented role in the metastatic progression of papillary thyroid carcinoma [79]. Members of the PDGF ligand family, including *PDGFA*, *PDGFB*, and *PDGFC*, are significantly upregulated in PTC compared to non-neoplastic thyroid tissue, further reinforcing the role of this pathway in tumor biology [44].

The loss of *c-KIT* expression is important in PTC [80], and masitinib demonstrated antitumor activity in c-KIT–negative hepatocellular carcinoma cells by inducing apoptosis through oxidative stress and JNK pathway activation, highlighting a promising alternative mechanism for cancer therapy [81]. The antineoplastic activity of masitinib in PTC identified by the DGIdb appears to be predominantly mediated through *PDGFRα* inhibition, a target with established relevance to PTC aggressiveness, making it a rational candidate for investigation in tumors overexpressing this receptor, particularly those with advanced or refractory lymph node disease.

Quizartinib is a second-generation inhibitor of class III receptor tyrosine kinases, acting on FLT3, KIT, and PDGFRα/β, with an excellent pharmacokinetic profile and high potency against mutant isoforms of these receptors [70].

In PTC specifically, FLT3 and c-KIT are part of a set of receptors with documented roles in thyroid neoplastic development, being targeted by multikinase inhibitors that have demonstrated antitumor activity in this context [82,83]. Recently, a Phase 3 clinical trial compared quizartinib plus standard chemotherapy versus placebo in patients with *FLT3-ITD*-positive acute myeloid leukemia and found that adding quizartinib significantly improved overall survival without a meaningful increase in toxicity, suggesting it is an effective and generally well-tolerated treatment option for this subtype of the disease [84].

The identification of quizartinib by the DGIdb as an antineoplastic agent relevant to PTC is therefore grounded in the shared molecular targets (*FLT3*, *KIT*, and *PDGFR*) whose expression has been documented in thyroid tumors, positioning this drug as a promising candidate for repurposing strategies, particularly in PTC with increased expression profiles of these kinases or in settings of resistance to currently approved targeted therapies.

### 4.5. Network Analysis Insights on PTC

These hub nodes likely represent critical regulators linking transcriptional and metabolic alterations in PTC. Norepinephrine is the biggest hub in the network, comprising 24 interactions. It can be related to promoting the Warburg effect, serving cancer as an alternative bioenergetics system [85], where cells oxidize glucose into lactic acid even with oxygen bioavailable for conventional glycolysis [86].

Additionally, arachidonic acid presented as an important member of the network, with 23 links. Inflammation is a well-known process fundamentally related to cancer progression [87]. Arachidonic acid is a well-known inflammatory and immune precursor molecule [88], and can be involved in several different processes in cancer once its enzymes (e.g., COX, LOX) are well studied [88], which were reported to be altered in other solid tumors [89].

Glutamic acid, on the other hand, is also important, with 21 connections. It is used as a carbon substrate for lipid synthesis and as a nitrogen donor [90]. In other work [91], it appears upregulated in PTC patients when compared to healthy volunteers, such as in this study, and it supposedly has effects in thyroid-stimulating hormone (TSH) production [92].

Additionally, the genes that link these hubs (norepinephrine, arachidonic acid, and glutamic acid), organized in Supplementary Table S9, show the tendency of centrality importance, but the relationships among them are poorly described and need more exploration.

The *SLC6A14* gene is presented as the most important of the genes in the network; it was found to be upregulated (as in this work) in most solid tumors [93], and its high concentration is an unfavorable prognostic indicator in other tumors. SLC6A14 is a solute transporter that supplies amino acids to the cell, supporting the growth and proliferation of cancer cells [94].

Other work using the same dataset found *ADK* in their analysis as a suggestive biomarker for PTC; here, we confirm this finding. In their work, ADK was found to participate in succinate metabolism and m6A modification of RNA, which could predict PTC [95]. Also, ADK was reported to convert extracellular adenosine to AMP [96], causing global hypermethylation [97,98].

Thus, the integrative network can provide novel insights into disease mechanisms and highlight potential targets for further investigation.

### 4.6. Limitations and Future Perspectives

The main limitation of this study concerns metabolomics data availability. A better approach would be to retrieve the metabolomics data, reprocess, and annotate the features. Unfortunately, this step of the process, besides being developed in-house, is only possible with the publication of metabolomics data in public repositories; only a few articles published the actual final feature table, making viable a sort of mining and integration of data by using a molecular network strategy.

On the other hand, the transcriptome datasets used have a reasonable but limited sample size, leading to unreliable findings. Due to sampling, it represents European people the most.

Hereafter, this pipeline will be tested with other cancers, integrating upstream with downstream data, preferably with an in vivo study design. The best scenario would be to perform an in-house RNA-seq and untargeted metabolomics analysis of the same samples to further explore and confirm these results. Additionally, a better way to integrate omics is not necessarily with molecular networks, even if it works, but with a well-designed experiment focused on multi-omics data merge. Arguably, molecular networking is still necessary as not everyone who has conducted studies have uploaded their data publicly to be systematically reviewed.

## 5. Conclusions

In this work, a methodology was demonstrated using publicly available data to intersect genes found in differential analysis (from 23,520 to 12) with the LASSO model (11 selected), achieving 11 selected genes. The intersection between DEGs and LASSO was a distinct technique to select the most relevant genes, as shown in the independent validation set curve (AUC = 91%, Sens. = 92%, Spec. = 97%, and Acc. = 95%). In addition, we performed survival analysis to assess the prognostic relevance of the 11 selected biomarkers, and four of them returned statistical differences, namely, *GLT1D1*, *PDZK1IP1*, *TENM1*, and *TIMP4*.

Merging different data types was possible due to network analysis, and with those interactions among metabolites and genes, biological discussion and insights were generated. Bioenergetics seems to be the main mechanism highlighted by this data, pointing to the Warburg effect as an important player behind PTC biology. In addition, inflammatory molecules, such as arachidonic acid and oleic acids, can regulate the inflammation status of the cancer, and once these molecules are a hub node in the network, the role of inflammation should be considered when describing PTC.

Drug–gene interactions were evaluated, and the observed result showed PDGFC-sunitinib and DPP4-nintedanib esylate, -masitinib, -quizartinib, and -olaratumab as ligand pairs. Afterward, molecular docking was performed to evaluate those findings; the binding energy demonstrates that the actual binding of the drug with the gene can happen, serving as a pointer regarding where to go in terms of therapeutic strategies.

Generally, this work has shown how well DEG–LASSO intersection performed, using multiple variables and restricting them to the most important ones. Complementary analyses were performed to further explore the potential of the genes as biomarkers. The network has shown a multi-layer integration of data types, bringing to light newer discussions in the PTC clinical context, such as the extent of the Warburg effect and inflammatory status, and novel competitors to biomarkers. 

## Figures and Tables

**Table 1:** Relevant statistical significance and predictive values of the eleven genes selected by this approach.

Genes	Average Expression	log2FC ^1^	Regulation	*p*-Value		Effect Size *	AUC ^2^	Sens.	Spec.	CI ^3^	
				Raw	Adjusted					Lower	Upper
*ABCA6*	4.16	−1.15	Down	3.15 × 10^−8^	4.28 × 10^−6^	0.6444	0.95	0.92	0.98	0.90	1.00
*ABHD17C*	5.92	1.16	Up	3.47 × 10^−9^	9.93 × 10^−7^	0.6772	0.96	0.94	0.98	0.91	1.00
*DPP4*	6.51	3.47	Up	4.32 × 10^−14^	6.79 × 10^−10^	0.7779	0.96	0.94	0.98	0.90	1.00
*GLT1D1*	5.38	1.67	Up	1.28 × 10^−12^	1.01 × 10^−8^	0.7850	0.86	0.754	0.87	0.79	0.94
*IGSF10*	4.52	−1.2	Down	3.50 × 10^−9^	9.93 × 10^−7^	0.7818	0.95	0.92	0.96	0.9	1.00
*PDGFC*	7.13	0.90	Up	2.76 × 10^−8^	3.92 × 10^−6^	0.3587	0.89	0.85	0.91	0.8	0.96
*PDZK1IP1*	4.98	2.27	Up	9.95 × 10^−10^	4.18 × 10^−7^	0.7810	0.95	0.96	0.91	0.91	1.00
*RHOU*	8.08	1.06	Up	8.30 × 10^−9^	1.65 × 10^−6^	0.6741	0.90	0.85	0.90	0.82	0.96
*RPS16P5*	6.50	−1.73	Down	1.52 × 10^−7^	1.26 × 10^−5^	0.3493	0.71	0.65	0.8	0.60	0.81
*TENM1*	4.81	2.23	Up	8.27 × 10^−12^	2.43 × 10^−8^	0.7857	0.87	0.71	0.98	0.79	0.95
*TIMP4*	3.93	−1.23	Down	6.52 × 10^−10^	3.39 × 10^−7^	0.6202	0.70	0.67	0.70	0.60	0.81

^1^: Log_2_ fold-change; ^2^: Area under the curve; Sens.: Sensitivity; Spec.: Specificity. ^3^: Confidence intervals. *: Small effect sizes were considered when the effect was <0.3, moderate effects were between 0.3 and <0.5, and finally, a large effect size was defined as values > 0.5.

**Table 2:** Biological process enrichment analysis.

Name	*p*-Value	False Discovery Rate
Cell adhesion	4.54 × 10^−21^	2.09 × 10^−17^
Extracellular matrix organization	1.40 × 10^−6^	3.22 × 10^−3^
Cell–cell adhesion	4.87 × 10^−7^	7.48 × 10^−3^
Homophilic cell–cell adhesion	9.61 × 10^−7^	1.11 × 10^−2^
Inflammatory response	1.54 × 10^−5^	1.42 × 10^−2^
Positive regulation of apoptotic process	3.30 × 10^−5^	2.14 × 10^−2^
Receptor-mediated endocytosis	3.66 × 10^−5^	2.14 × 10^−2^
Nervous system development	3.70 × 10^−5^	2.14 × 10^−2^
Ossification	1.02 × 10^−4^	5.20 × 10^−2^
Positive regulation of phosphatidylinositol 3-kinase/protein kinase B signal transduction	1.39 × 10^−4^	6.39 × 10^−2^

**Table 3:** Connections of the top three genes based on the degree.

Gene	Connects to
*SLC6A14*	Glycine, cysteine, alanine, lysine, methionine, isoleucine, beta-alanine, and leucine
*ADK*	Aspartic acid, adenine, guanosine, hypoxanthine, aminolevulinic acid, inosine, creatine, and guanidoacetic acid
*ATIC*	Aspartic acid, adenine, hypoxanthine, citric acid, and inosine

## Data Availability

Data supporting the findings in this study are included within the Supplementary File. Gene expression data analyzed during the current study are available in the Gene Expression Omnibus (GEO) repository under persistent accession numbers GSE29265 and GSE33630. Metabolites were collected from [99,100,101].

## Supplementary Materials

The following supporting information can be downloaded at: https://www.mdpi.com/article/10.3390/biology15131004/s1, Figure S1: Complete gene-metabolite network displaying in squared shapes the metabolites, and in oval the genes. Colors are mapped to the fold–change values; Table S1: All the Differential Expressed Genes and their statistical values; Table S2: LASSO coefficients different than zero; Table S3: Genes and metabolites and their main function in cancer; Table S4: Overall Survival Analysis results of the selected genes; Table S5: Progression-free Intervals from the selected genes; Table S6: Enrichment Analysis of the DEGs (FDR < 0.05); Table S7: Drug-gene interaction table; Table S8: Metabolites table comprising all used metabolites with respective *p*-values, fold-change and PLS-DA’s VIP score; Table S9: Genes that link the hub metabolites and its function; Table S10: Over Representation Analysis of the metabolites; Table S11: Input list used to perform some of the analysis; Table S12: Pathways in which the selected genes play a row based on KEGG; Table S13: Prediction evaluation of each of the selected genes; Table S14: All scores generated in the network analysis, ranking the metabolites and genes.

## Abbreviations

The following abbreviations are used in this manuscript:

TC	Thyroid Carcinoma
PTC	Papillary Thyroid Carcinoma
FTC	Follicular Thyroid Carcinoma
MTC	Medullary Thyroid Carcinoma
TSH	Thyrostimulating Hormone
ATA	American Thyroid Association
GEO	Gene Expression Omnibus
FDR	False Discovery Rate
LASSO	Least Absolute Shrinkage and Selection Operator
ROC	Receiver Operating Characteristic
DEG	Differentially Expressed Genes
PCA	Principal Component Analysis
AUC	Area Under the Curve
TCGA	The Cancer Genome Atlas
DAVID	The Database for Annotation, Visualization, and Integrated Discovery
DPP4	Dipeptidyl Peptidase 4
GLT1D1	Glycosyltransferase 1 Domain Containing 1
TENM1	Teneurin Transmembrane Protein 1
TIMP4	TIMP Metallopeptidase Inhibitor 4
PDZK1IP1	PDZK1 Interacting Protein 1
ABHD17C	Abhydrolase Domain Containing 17C, Depalmitoylase
IGSF10	Immunoglobulin Superfamily Member 10
RHOU	Ras Homolog Family Member U
PDGFC	Platelet-Derived Growth Factor C
ABCA6	ATP Binding Cassette Subfamily A Member 6
RPS16P5	Ribosomal Protein S16 Pseudogene 5
RAS	RAS GTPase
MAPK	Mitogen-activated Protein Kinase
COX	Cyclooxygenase
LOX	Lipooxygenase
iPLA2β	Calcium-independent phospholipase A2β
SLC6A14	Solute Carrier Family 6 Member 14
ADK	Adenosine Kinase
RNA	Ribonucleic acid
AMP	Adenosine monophosphate
SYT1	Synaptotagmin 1
CYSLTR2	Cysteinyl Leukotriene Receptor 2
PPARG	Peroxisome Proliferator Activated Receptor Gamma
SLC4A4	Solute Carrier Family 4 Member 4
CYCS	Cytochrome C, Somatic
JUN	Jun Proto-Oncogene, AP-1 Transcription Factor Subunit
