# Supplementary material for: Exploratory Machine Learning and Omics Integration in the Search for Biomarkers of Papillary Thyroid Cancer
Source: Biology (Basel). 2026 Jun 25;15(13):1004. doi: 10.3390/biology15131004 (PMC13359534; doi:10.3390/biology15131004)
Supplement: Supplementary file 1 [file biology-15-01004-s001.zip › Supplementary Figure S1.pdf]

Exploratory Machine Learning and Omics Integration in the Search for Biomarkers of Papillary Thyroid Cancer

Pedro Henrique Godoy Sanches, Nicolly Clemente de Melo, Danilo Cardoso de Oliveira and Lucas Miguel de Carvalho *

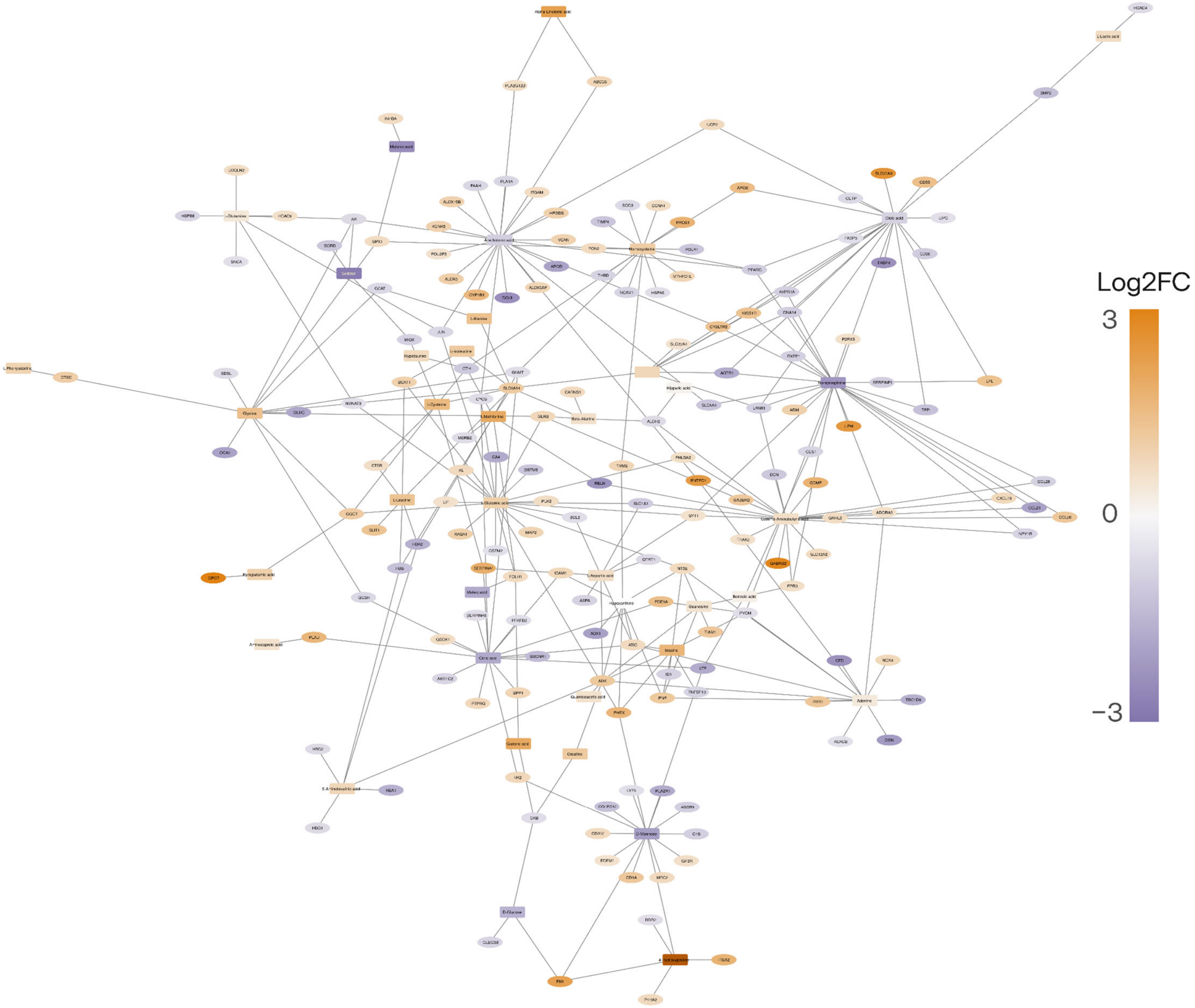

Supplementary Figure S1. Complete gene–metabolite network displaying in squared shapes the metabolites, and in oval the genes. Colors are mapped to the fold–change values.
